# A new route to the nucleus

**DOI:** 10.7554/eLife.83308

**Published:** 2022-10-13

**Authors:** Hongyu Bao, Hongda Huang

**Affiliations:** 1 Key Laboratory of Molecular Design for Plant Cell Factory of Guangdong Higher Education Institutes, Department of Biology, School of Life Sciences, Southern University of Science and Technology Shenzhen China

**Keywords:** histone, nuclear import, importin, histone chaperone, chromatin, Human

## Abstract

A newly discovered pathway suggests histone proteins H3 and H4 are imported into the nucleus as individual units rather than joined together as heterodimers as was previously thought.

**Related research article** Pardal AJ, Bowman AJ. 2022. A specific role for importin-5 and NASP in the import and nuclear hand-off of monomeric H3. *eLife* **11**:e81755. doi: 10.7554/eLife.81755.

The genetic material of animals, yeast and other eukaryotes is organized into a tightly wound structure known as chromatin which is stored inside the nuclei of cells. Chromatin is organized into subunits called nucleosomes that are made up of small segments of DNA wrapped around eight histone proteins: two copies of the protein H3 which each dimerize with an H4 protein, and two H2A proteins which each dimerize with another protein called H2B (Figure 1A; Luger et al., 1997). The two H3-H4 heterodimers then attach to one another to form a tetramer.

**Figure 1.**
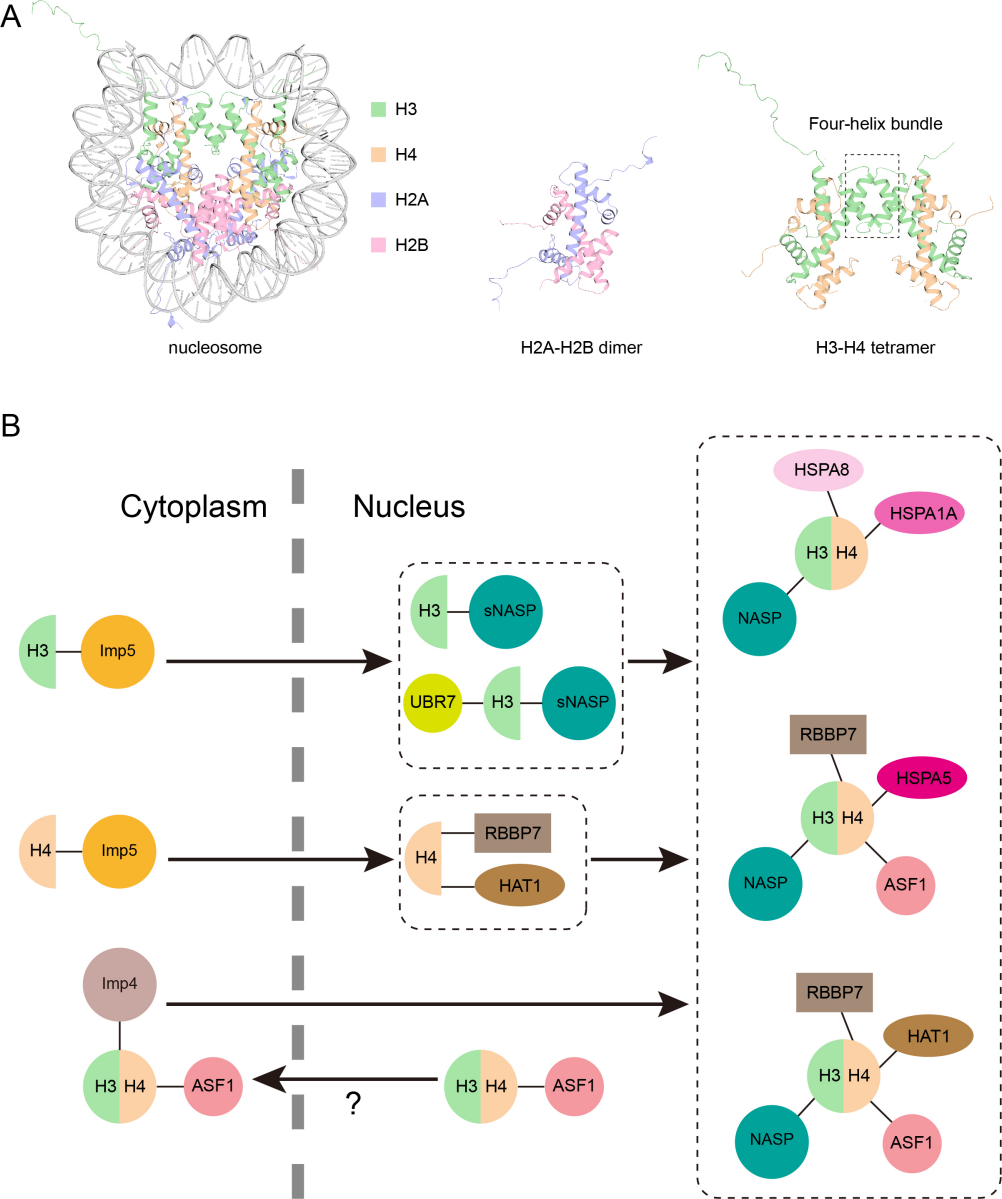
Model of the new pathway that imports histone proteins H3 and H4 into the nucleus. (**A**) Three-dimensional structure of the nucleosome, a segment of DNA (grey) wrapped around eight histone proteins: two copies of H3 (green), H4 (orange), H2A (purple) and H2B (pink). H2A and H2B join together in a head to tail configuration to form a heterodimer (middle). H3 and H4 also connect in the same way, and the two heterodimers attach to one another via their helices (outlined by the box) to form a tetramer (right). Structures from PDB ID: 1AOI. (**B**) Pardal and Bowman have found a new pathway through which monomers of H3 and H4 are imported from the cytoplasm into the nucleus (black arrow) via the protein Imp5 (yellow circle; top left). Once inside the nucleus, H3 and H4 are transferred to a version of the NASP protein (known as sNASP) and the HAT1–RBBP7 complex, respectively, which together with other proteins help the two monomers dimerize (top middle). The H3-H4 heterodimer is then folded in the nucleus and assembled into one of three complexes which include different chaperone proteins (shown in pink; right). Previously it was thought that H3-H4 heterodimers were formed in the cytoplasm before being imported to the nucleus via a pathway mediated by Imp4 (mauve circle) and ASF1 (pink circle; bottom left). However, it is possible that this H3-H4-ASF1 complex detected in the cytoplasm may have leaked out from the nucleus, meaning the pathway discovered by Pardal and Bowman may be the dominant route H3 and H4 take in to the nucleus.

Image credit: Hongyu Bao (CC BY 4.0).

In order to divide, cells must replicate their DNA and histone proteins so that the genetic material that will be passed to their daughter cells can be assembled into chromatin. Half of the H3 and H4 histones that go in to the chromatin of each daughter cell are recycled from old tetramers and heterodimers, while the rest are newly synthesized structures. Freshly made H3 and H4 histones are also incorporated into chromatin throughout a cell’s lifetime during various DNA-related processes, like transcription.

For decades it was assumed that newly synthesized H3 and H4 histones dimerize in the cytoplasm and are then transported into the nucleus by two proteins: importin-4 (Imp4) and the histone chaperone ASF1 (Grover et al., 2018; Pardal et al., 2019; Bernardes and Chook, 2020). This nuclear import model was supported by previous experiments that found H3-H4 heterodimers in samples taken from the cytosol of cells. However, a study in 2018 suggested that preparation of these extracts may have ruptured the nuclear membrane, allowing H3-H4 heterodimers to leak out of the nucleus into the cytosol (Apta-Smith et al., 2018). This led the researchers to conclude that H3 and H4 may be transported into the nucleus as individual units or monomers. Now, in eLife, Alonso Pardal and Andrew Bowman from the University of Warwick – who are part of the research group that conducted the 2018 study – report new findings confirming this theory (Pardal and Bowman, 2022).

The nuclear import, dimerization, and incorporation of histone H3 and H4 into chromatin occur very rapidly, making it difficult to track each individual step. To overcome this issue, Pardal and Bowman designed H3 and H4 proteins which are unable to dimerize, and transiently expressed these mutant proteins in cells cultured in the laboratory. They found that both mutants were still imported into the nucleus, but were not incorporated into chromatin. This allowed Pardal and Bowman to work out which proteins the H3 and H4 monomers interact with inside cells.

Intriguingly, instead of binding to Imp4, both histones attached to Imp5, which suggests that they primarily associate with Imp5 when they are not bound to each other. Once in the nucleus, the mutants also interacted with distinct histone chaperone proteins: H3 monomers associated with a protein called NASP, while H4 monomers interacted with a protein complex called HAT1–RBBP7 (Figure 1B).

Next, Pardal and Bowman carried out further experiments to see how NASP and Imp5 cooperate when H3 is imported into the nucleus. This revealed that NASP was bound to at least twice as many wildtype H3 molecules as wildtype H4 molecules, indicating that there is a sub-pool of H3 monomers attached to NASP proteins. Additional biochemical experiments and analyses showed that NASP forms five distinct complexes with histones: two complexes that contain monomeric H3, and three that contain the H3-H4 heterodimer plus various chaperone proteins (Figure 1B). However, Imp5 was not detected in any of these NASP complexes, indicating that H3 cannot be bound to NASP and Imp5 simultaneously. This was supported by further in vitro experiments showing H3 being transferred from Imp5 to NASP.

Finally, Pardal and Bowman investigated how H3, Imp5 and NASP interact with one another in living cells using two techniques called mito-F2H and RAPID-release (Bowman et al., 2016; Apta-Smith et al., 2018). This revealed that there were 100 times more Imp5 proteins interacting with cytoplasmic H3 than Imp4 molecules, confirming that Imp5 is the major protein importing H3 into the nucleus. Once the Imp5-bound H3 monomer enters the nucleus, a version of the NASP protein (known as sNASP) immediately sequesters H3. Although Pardal and Bowman had not fully characterized the H4 monomers bound to HAT1-RBBP7, they propose that monomeric H4 enters the nucleus and is incorporated into chromatin in a similar way.

In summary, this research identifies a new pathway for importing histone proteins: newly synthesized H3 and H4 are transported into the nucleus as monomers by the protein Imp5, and are then handed off to NASP and the HAT1–RBBP7 complex in the nucleus, respectively (Figure 1B). Together with other proteins, NASP and HAT1–RBBP7 help H3 and H4 dimerize so they can be assembled into chromatin to compact DNA.

However, it is still unclear how this newly discovered route relates to the pathway that scientists have long thought was responsible for importing H3-H4 heterodimers. Further work will help to answer this question and shed light on the metabolism of histone proteins H3 and H4.
